# Patterns of cervical cytological abnormalities according to the Human Development Index in the northeast region of Brazil

**DOI:** 10.1186/s12905-016-0334-2

**Published:** 2016-08-12

**Authors:** José De Ribamar Pinho-França, Maria Bethânia Da Costa Chein, Luiz Claudio Santos Thuler

**Affiliations:** 1 Department of Medicine III, Federal University of Maranhão (Universidade Federal do Maranhão), Praça Gonçalves Dias, 21/2° andar, Centro, São Luís, MA 65020-240 Brazil; 2 Graduate Program in Oncology, National Cancer Institute (Instituto Nacional de Câncer), Rua André Cavalcanti, 37/2° andar, Centro, Rio de Janeiro, 20231-050 Brazil

**Keywords:** Cervical cancer, Human development index, Health status disparities, Cytological Abnormalities, Cancer precursor lesions, Brazil

## Abstract

**Background:**

Disparities in cancer incidence and mortality rates between regions arise due to differences in socioeconomic conditions and in human development factors. The major purpose of this study was to measure the role of the Human Development Index (HDI) in the pattern of cervical cytological abnormalities (CCAs).

**Methods:**

This was an analytical sectional study involving a review of secondary cervical cytology data collected from women living in the state of Maranhão, Brazil, in 2007–2012 and collected from the Cervical Cancer Information System (Sistema de Informação do Câncer do Colo do Útero - SISCOLO). The cervical screening results were classified according to the Brazilian Classification of Cervical Reporting (Nomenclatura Brasileira para Laudos Cervicais), an adaptation of the Bethesda System. The Municipal Human Development Index (MHDI) was used, which is an adaptation of the global HDI. The association between CCAs and MHDI was evaluated using the chi-squared test and odds ratios (ORs) with 95 % confidence intervals (95 % CI). The significance level used for all tests was 5 %.

**Results:**

We analysed 1,363,689 examinations of women living in the state of Maranhão. CCAs were identified in 2.0 % of smears in municipalities with high MHDI, 2.2 % in those with medium or low MHDI and 4.1 % in those with very low MHDI. In addition, potentially malignant changes and suspected cervical cancer (HSIL+) were 40.0 % more frequent (0.3 %) in municipalities with medium or low MHDI and 3.6 times more frequent (0.8 %) in municipalities with very low MHDI compared to those with high MHDI (0.2 %).

**Conclusion:**

The association between MHDI and the occurrence of CCAs and HSIL+ shows that more developed areas with more effective health services have a lower prevalence of these lesions. To control cervical cancer, it is necessary to reduce social inequality and improve the availability of health services.

## Background

Cervical cancer is the fourth-most common cancer in women, with 528,000 new cases annually and 266,000 deaths in 2012. It has become a major public health problem [1]. In Brazil, the estimate for 2016 is 16,340 new cases with an risk of 15.85 cases per 100,000 women, which would make it the third-most common cancer in the female population. It contributes to the high female mortality rate in the country, and in some regions, it is the leading cause of cancer death [2]. For the state of Maranhão, located in the northeast of Brazil, it has been estimated that in 2016, cervical cancer will be the most common gynaecological cancer, with 28.57 new cases per 100,000 women, one of the highest estimated incidence rates in Brazil. It is important to highlight the real possibility of underestimation, especially in municipalities other than the capital of the state, which historically tend to underreport data, as 23.7 % of expected cases are found in the capital São Luís, with an estimated crude incidence rate of 42.58/100,000 women [2]. In addition, the mortality rate for this cancer adjusted for the world population in 2012 (last year with available data) was 10.22 per 100,000 women, and it remained above 9.00 per 100,000 women between 2005 and 2011 [3].

In Brazil, cervical cancer screening programs are based on Pap smear testing. Smears are read in a large number of laboratories by certain professional groups such as physicians (cytopathologists and pathologists), pharmacists, biologists, biomedical scientists or cytotechnologists. Defined standards and criteria are used to measure the quality of cytopathology testing and to assess the performance of public and private laboratories providing services to the Brazilian Unified Health System (Sistema Único de Saúde – SUS).

The high incidence and mortality, principally in less developed regions [1], reveals inequalities between different socioeconomic groups and exposure to risk factors present in these areas. Its occurrence stems from poor living conditions [4], such as a lack of, or difficulty of access to, health services, especially population screening programs [5]. From this perspective, the United Nations Human Development Index (HDI) is a good indicator for better understanding how these underlying causes may contribute to differences in the geographical and temporal distribution of risk factors and to determine occurrence patterns of this cancer. This is justified by the fact that the HDI, a summary measure of human development, incorporates primordial human development dimensions such as long and healthy life (based on life expectancy at birth), access to knowledge (based on a combination of adult literacy rate and primary education to tertiary education enrolment rates) and decent living standards (based on Gross National Product - GDP per capta adjusted for purchasing-power parity). In that regard, it can be seen as an indirect indicator of adverse living conditions, given that 69.7 % of new cases and 74.9 % of deaths from cervical cancer occur in less developed countries with medium or low HDI [1]. Following this trend, mortality from this cancer in Brazil is more frequent in socio-economically disadvantaged regions [6, 7]. In the highest HDI regions of the world, the cancers of the female breast, lung, colorectum, and prostate were the most common, whereas in medium HDI regions, the cancers of the oesophagus, stomach, and liver were the predominant forms, and in low HDI regions, cervical cancer was more common [8]. Against this backdrop, and considering that the prevention and control of cervical cancer is predicated on routine cervical screening, a strategy known to be effective in fighting the problem, it is important to know the profile of cervical screening conducted in the state of Maranhão. The results can contribute to the adoption of public policies with greater efficiency and that are more locally appropriate in combating a disease that at least in theory should be easily avoidable. This will enable us to qualify the situational diagnosis and to create interventions that increase screening efficiency. Although the state of Maranhão occupies the penultimate place in the HDI ranking of Brazilian states, there is heterogeneous resource distribution, and living condition indicators vary widely among its municipalities [9]. This study is therefore justified in order to identify, for the first time in a Brazilian state, the role of the Human Development Index (HDI) in the pattern of CCAs.

## Methods

This was an analytical sectional study with a review of secondary cervical cytology data performed on women living in the state of Maranhão in 2007–2012 and collected from the Cervical Cancer Information System (Sistema de Informação do Câncer do Colo do Útero - SISCOLO). This system was established by the Ministry of Health of Brazil in 1999 to assist in the reimbursement of exams and monitoring and evaluation of the Brazilian Cervical Cancer Early Detection Program (Programa Nacional de Controle do Câncer de Colo do Útero - PNCCCU) activities. The database was made available electronically by the Health Secretariat of the State of Maranhão. The data, originally organised into monthly files, were exported into a *Data Base File* (DBF) format and aggregated into a single database in Microsoft Excel 2010. From a total of 1,368,891 cervical screenings, 5,202 (0.4 %) identified as a “rejected sample” were excluded.

The variables analysed were age, place of residence, previous cervical screening and time since last cervical screening. The cervical screening results were classified according to the Brazilian Classification of Cervical Reporting [10], an adaptation of the Bethesda System adopted in the country since April 2006 on the recommendation of the Ministry of Health. The smears were analysed regarding adequacy of the sample, presence or absence of the transformation zone (TZ) component and presence of atypical cells. In this study, the presence of an adequate number of metaplastic and/or glandular epithelia on the examined material were considered representative of TZ epithelia [11]. The examinations were classified as within normal limits, benign cellular changes (inflammation, repair, immature squamous metaplasia, atrophy with inflammation, radiation, etc.) or atypical cells. Atypical cells were classified as atypical cells of undetermined significance (atypical squamous cells [ASC], atypical glandular cells [AGC] or of unknown origin), atypical squamous cells (low-grade squamous intraepithelial lesion [LSIL], comprising cytopathic effect by human papillomavirus [HPV] and grade I cervical intraepithelial neoplasia; high-grade squamous intraepithelial lesion [HSIL], including grades II and III cervical intraepithelial neoplasia; HSIL not excluding micro-invasion; squamous cell carcinoma [SCC]); atypical glandular cells (adenocarcinoma in situ [AIS]; and invasive adenocarcinoma [IA]). Potentially malignant changes and suspected cervical cancer (HSIL, SCC, AIS e IA) were grouped as HSIL+. The Municipal Human Development Index (MHDI) for the woman’s place of residence was used. The MHDI is an adaptation of the global HDI, adjusted for national indicators [9]. The MHDI adjusts the HDI to the municipal reality and reflects specific and regional challenges in Brazilian human development. The MHDI assesses the same dimensions as the global HDI – health, education and income – however, some of the applied indicators are different. The MHDI also varies between 0 (minimum value) and 1 (maximum value). The municipalities were classified as high MHDI (0.700 to 0.799), medium MHDI (0.600 to 0.699), low MHDI (0.500 to 0.599) and very low MHDI (0.400–0.499). Medium and low MHDI were grouped into a single category based on their distribution in regard to other study variables.

Statistical analysis was performed using IBM SPSS Statistics software, version 20. The chi-squared test was applied to compare categorical variables. Odds ratios (OR) and their respective 95 % confidence intervals (95 % CI) were also calculated. The significance level used for all tests was 5 %.

To estimate failure in the detection process due to a lack of TZ-representative cellular elements, the number of unidentified cervical cancer and precursor lesions was calculated according to the following Eq:

$$ plpc{a}^{ni}=\left[\left(O{R}^{tz}\times lpc{a}^{i^{s\;/\;tz}}\right)-\left(lpc{a}^{i^{\;s\;/\;\mathrm{t}\mathrm{z}}}\right)\right] $$

Where *plpca*^*ni*^ represents the number of non-identified potentially malignant changes and suspected cervical cancer; *OR* ^*tz*^ represents the OR found regarding the association of precursor and cervical cancer-suspicious lesions among the groups with smears with and without TZ representativeness; and $lpc{a}^{i^{\;s/\;tz}}$ represents the total number of cervical cancer and precursor lesions detected in smears with no TZ cells.

The research project was approved by the Research Ethics Committee of the Federal University of Maranhão N. 045181/2013, as per the National Council of Health Resolution number #466/2012. Written informed consent was not required due to the observational nature of the study.

## Results

We analysed 1,363,689 samples of women living in the state of Maranhão. The capital of the state, São Luís, contributed with 21.5 % of patients; 68.7 % were from municipalities with medium or low MHDI, 31.0 % from municipalities with high MHDI and 0.3 % from municipalities with very low MHDI. The age varied from 12 to 99 years, with 76.3 % of tests in women aged 25 to 64 years, 19.9 % inwomen under the age of 25 years and 3.8 % inwomen aged over 65 years. Tests were being performed for the first time in 16.9 % of cases. Previous cervical screening was reported by women in 61.0 % of cases. Of these, 65.5 % were performed after an interval since the previous test of up to 3 years (Table 1).

**Table 1:** Sociodemographic characteristics of women participating in the study. State of Maranhão, Brazil. 2007 to 2012

Variable	N	%
Age group (years)		
<25	271.279	19.9
25 to 29	214.668	15.7
30 to 34	195.841	14.4
35 to 39	165.181	12.1
40 to 44	140.481	10.3
45 to 49	119.722	8.8
50 to 54	95.378	7.0
55 to 59	67.114	4.9
60 to 64	42.102	3.1
>64	51.923	3.8
Prior preventive exam		
Never done	231.132	16.9
Done previously	832.322	61.0
Missing	300.235	22.0
Time since last exam^a^		
≤ 1 year	342.362	41.1
2 years	147.032	17.7
3 years	55.724	6.7
> 3 years	48.708	5.9
Missing	238.496	28.7
Place of residence		
Capital	292.919	21.5
Other municipalities	1.070.770	78.5
MHDI of woman’s place of residence		
High	423.202	31.0
Medium or low	936.038	68.7
Very low	4.449	0.3
Total	1.363.689	100.0

^a^Only for those who already had prior screening

*MHDI* Municipal Human Development Index

Among the 1,327,691 tests considered satisfactory for cytological evaluation (97.4 % of total), only 58.8 % had TZ-representative epithelia, 2.2 % had atypical cells, and the rest (97.8 %) were characterised as within normal limits or with benign cellular changes. Atypical cells were identified in 2.0 % of smears in municipalities with high MDHI, 2.2 % in those with medium or low MHDI and 4.1 % in those with very low MHDI. Atypical squamous cells of undetermined significance were identified in 1.1 % of cytological smears, LSIL in 0.8 % and HSIL in 0.3 %. Atypia suggestive of HSIL+ were observed in 0.3 % of all analysed tests. The percentage of satisfactory smears in municipalities with high, medium or low and very low MHDI was 98.2, 97.0 and 98.8 %, respectively. However, TZ representativeness, the prevalence of atypical cells and HSIL+ prevalence were inversely proportional to MHDI (Table 2).

**Table 2:** Cervical cytopathology exams performed in the state of Maranhão, Brazil. 2007 to 2012

Variables	High MHDI		Medium or low MHDI		Very low MHDI		Total	
	N	%	N	%	N	%	N	%
Sample adequacy								
Unsatisfactory	7,565	1.8	28,378	3.0	55	1.2	35,998	2.6
Satisfactory	415,637	98.2	907,660	97.0	4,394	98.8	1,327,691	97.4
Transformation zone								
Absent	175,332	57.8	370,796	40.9	879	20.0	547,007	41.2
Present	240,305	42.2	536,864	59.1	3,515	80.0	780,684	58.8
Cervical screening diagnosis^a^								
Within normal limits or with benign abnormalities	407,253	98.0	887,565	97.8	4,214	95.9	1,299,032	97.8
Cellular atypia^a^	8,384	2.0	20.095	2.2	180	4.1	28,659	2.2
Atypical cells of undetermined significance^a^	3,005	0.7	11,433	1.3	49	1.1	14,487	1.1
Low-grade squamous intraepithelial lesion (LSIL)^a^	4,429	1.1	5,735	0.6	95	2.2	10,259	0.8
High-grade intraepithelial lesion or worse (HSIL+)^a^	950	0.2	2,927	0.3	36	0.8	3,913	0.3

^a^Only for satisfactory samples

*MHDI* Municipal Human Development Index, *TZ* transformation zone

**Fig. 1:**
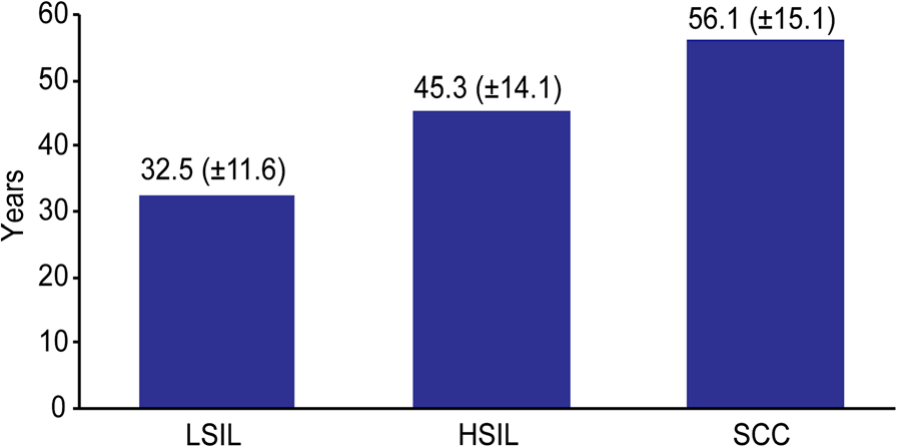
Mean age (±Standard Deviation) according to the citological anormality. State of Maranhão, Brazil. 2007 to 2012

The women’s mean age was 36.8 (±13.6) years. There was an age variation according to the citological anormality: 32.5 years (± 11.6) for cases of LSIL, 45.3 years (± 14.1) for cases of HSIL and 56.1 years (± 15.1) for cases suspected of SCC (Fig. 1).

The chance of detecting any atypia increased three times when there was TZ representativeness and by 20.0 % when the woman reported having been screened previously or when time since the last test was more than three years (Table 3). The chance of detecting LSIL increased by 3.8 times with TZ representativeness in the smear, increased by 20.0 % when the last examination was more than 3 years ago and decreased by 10.0 % in women who had had a prior examination (Table 3). The chance of detecting HSIL+ increased 2.7 times when there was TZ representativeness and 20.0 % when the last examination was more than 3 years ago and when the woman had been screened previously. Using the PNCCCU prioritary age group as a reference, i.e., age 25 to 64, there was a decrease of 10.0 and 80.0 %, respectively, in the chance of detecting any atypia and detecting HSIL+ in the age group less than 25 years and, at the same time, an increase of 50.0 % in the chance of detecting LSIL in the same age group. On the other hand, for the ≥65-year age group, there was a 50.0 % decrease in the chance of detecting LSIL, while there was an increase of 40.0 and 180 %, respectively, in the chance of detecting any atypia or HSIL+ (Table 3).

**Table 3:** Variables associated with the presence of any atypia, LSIL or HSIL+ in cervical cytopathology exams. State of Maranhão, Brazil. 2007 to 2012

Variables	Yes		No		OR	95 % CI	*p*-value
	n	%	n	%			
Any Atypia							
Age < 25 years vs. 25 to 64 years	5,186	2.0	260,215	98.0	0.9	0.89–1.95	<0.001
Age ≥ 65 years vs. 25 to 64 years	1,467	3.0	48,005	97.0	1.4	1.34–1.49	<0.001
Presence vs. absence of TZ	22,615	2.9	758,069	97.1	3.0	2.86–3.03	<0.001
Already had prior exam vs. Never had	16,896	2.0	815,426	98.0	1.2	1.11–1.19	<0.001
Time since last screening > 3 years vs. ≤ 3 years	1,127	2.3	47,581	97.7	1.2	1.10–1.24	<0.001
LSIL							
Age < 25 years vs. 25 to 64 years	2,881	1.1	262,520	98.9	1.5	1.47–1.60	<0.001
Age ≥ 65 years vs. 25 to 64 years	172	0.3	49,300	99.7	0.5	0.43–0.58	<0.001
Presence vs. absence of TZ	8,651	1.1	772,033	98.9	3.8	3.60–4.02	<0.001
Already had prior exam vs. Never had	5,237	0.6	827,085	99.4	0.9	0.86–0.96	0.001
Time since last screening > 3 years vs. ≤ 3 years	361	0.7	48,347	99.3	1.2	1.03–1.28	0.015
HSIL+							
Age < 25 years vs. 25 to 64 years	157	0.1	265,244	99.9	0.2	0.16–0.22	<0.001
Age ≥ 65 years vs. 25 to 64 years	438	0.9	49,034	99.1	2.8	2.51–3.07	<0.001
Presence vs. absence of TZ	3,043	0.4	777,641	99.6	2.7	2.48–2.90	<0.001
Already had prior exam vs. Never had	2,305	0.3	830,017	99.7	1.2	1.06–1.27	0.002
Time since last screening > 3 years vs. ≤ 3 years	163	0.3	48,545	99.7	1.2	1.04–1.44	0.016

*OR* odds ratio, 95 *% CI*, 95 % confidence interval, *TZ*, transformation zone

*LSIL* Low-grade squamous intraepithelial lesion, *HSIL+* high-grade squamous intraepithelial lesionor worse

The stratified analysis by age group showed that women between 25 and 64 years (OR = 0.9) and those ≥ 65 years (OR = 0.8) were less likely to have cervical cancer and precursor lesions when they had had previous cervical screening. In contrast, in women under 25 years of age, having undergone previous screening increased the chance of detection of these lesions by 60 % compared to women who had not been screened previously (OR = 1.6) (Table 4). The number of unidentified cervical cancer and precursor lesions was estimated for 1,357 cases due to a lack of TZ-representative cellular elements. Finally, the chance of detection of cervical cancer and precursor lesions increased by 40.0 % in municipalities with medium or low MHDI and 3.6 times in municipalities with very low MHDI compared to those with high MHDI (Table 5).

**Table 4:** Association between having had prior screening and the presence of HSIL+ (squamous or glandular) stratified by age group. State of Maranhão, Brazil. 2007 to 2012

Had prior screening	Yes		No		OR	95%CI	*p*-value
	n	%	n	%			
< 25 years							
No	35	0.0	87,362	100.0	Reference		
Yes	74	0.1	113,252	99.9	1.6	1.09–2.44	< 0.001
25–64 years							
No	436	0.3	129,731	99.7	Reference		
Yes	1,992	0.3	665,237	99.7	0. 9	0.80–0.98	< 0.001
≥65 years							
No	81	1.1	7,570	98.9	Reference		
Yes	239	0.8	29,502	99.2	0.8	0.59–0.97	< 0.001

*OR* odds ratio, 95*%CI* 95 % confidence interval

**Table 5:** Association between Municipal Human Development Index (MHDI) and the presence of HSIL+ (squamous or glandular). State of Maranhão, Brazil. 2007 to 2012

MHDI	Yes	%	No	%	OR	95 % CI	*p*-value
High	930	0.2	414.707	99.8	Reference		
Medium or low	2.875	0.3	904.785	99.7	1.4	(1.30–1.50)	< 0.001
Very low	36	0.8	4.358	99.2	3.6	(2.65–5.17)	< 0.001

*OR* odds ratio, 95 *% CI* 95 % confidence interval

*MHDI* Municipal Human Development Index

## Discussion

The profile of the study population was women who spontaneously approached the public health system (SUS) seeking cervical screening. The majority lived in municipalities with medium or low MHDI (68.7 %), although the capital, São Luís, with the highest index in the state, contributed to a considerable number of cases (21.5 %). The results of this study showed that there was a close association between MHDI and detection of cervical cancer and precursor lesions. To our knowledge, this is the first study to investigate this association.

The high demand for tests by women in the 25-64-year age group (76.3 %), which is the age group at the highest risk for cervical cancer, is in keeping with the Brazilian Guidelines for Cervical Cancer Screening [11], which recognises this as the target population of the screening program. In line with this recommendation, 84.5 % of cervical cancer and precursor lesions were described in this age group. A study conducted in 2011 in two Brazilian municipalities with different socioeconomic characteristics, Rio de Janeiro in the southeast region and Maceió in the northeast region, showed that the percentage of tests in the 25–64 year age group was 78.1 and 74.7, respectively, with a predominance of younger women in Rio de Janeiro and older in Maceió [12]. Another study conducted in the state of Maranhão analysing the 139,505 cervical screenings performed during 2011 showed that 76.8 % were women aged 25–64 [13].

In accordance with the recommendations from the Brazilian Guidelines [11], this study showed that in most cases (58.8 %), women have exams at intervals of less than three years. Higher rates were observed in Maceió (68.8 %) and Rio de Janeiro (74.3 %) [12]. In another study involving women living in 13 communities located in Baixada Fluminense, on the outskirts of the state capital of Rio de Janeiro, 70.7 % of women had up-to-date screening [14]. Note that annual repetition of the examination, which occurred in 41.1 % of cases, does not increase the protective effect of screening [15]. At the other extreme, when the recommended three-year interval was exceeded, there was an increase of 17.0 % in the chance of presenting atypia, 15.0 % of LSIL and 22.0 % of HSIL+. These data regarding age and frequency of cervical screening indicate irregular access to tests and an overloading of the health care system with unnecessary consumption of resources.

The percentage of unsatisfactory smears for cytological evaluation in this study (2.6 %) is within the minimum quality standard established by the Ministry of Health, which is up to 5.0 % [16]. Similar figures were observed in a study conducted in the city of Goiânia, located in the Midwestern region of the country, in which, from a total of 10,951 smears, 2.3 % were classified as unsatisfactory [17]. However, this is well above the percentage found in Brazil, in 2002, in a study involving 739 laboratories and 10,505,773 cervical cytology screenings, of which 1.6 % were considered unsatisfactory; 25.0 % of the laboratories analysed (25th percentile) had more than 2.0 % of unsatisfactory samples [18]. More recently, a profile analysis of cervical-uterine cytology laboratories showed that of 10,275,476 tests performed in the Brazilian public health system in 2010, only 1.0 % were considered unsatisfactory [19]. In Maceió and Rio de Janeiro the percentages of unsatisfactory examinations were 0.4 and 0.2 %, respectively [12].

Several studies have confirmed the association between TZ epithelial representativeness, an indicator of specimen collection quality, and the detection of cytological abnormalities, and an association between the lack of these cells and limitations in sample analysis with high rates of false-negative results [17, 20]. In this study TZ representativeness proved to be very weakened in that only 58.8 % of the collected samples showed it. In fact, the results presented here indicate a greater chance of identifying any atypia (OR = 3.0), LSIL (OR = 3.8) and HSIL+ (OR = 2.8) in the presence of TZ-representative cell groups. In São Paulo, a review of 24,316 cervical screening reports conducted between January 2005 and December 2008 in a single pathology laboratory showed that among patients who were younger than 40 years, 88.8 % had TZ material in their smears, as opposed to only 53.0 % of those aged 40 or more [21]. A lack of TZ representativeness has contributed, at least in part, to the low sensitivity of the screening process in detecting citological abnormalities in the population examined in the present study, and this was mirrored by the positivity rate. This result allowed us to estimate that the screening program during this period failed to identify 26.1 % (1,357/5,198) of HSIL+ lesions. Note that it is precisely on the basis of correct identification of potentially malignant lesions that the screening program can reduce the incidence and mortality from this cancer. Thus, the low TZ representativeness identified, despite not having been corrected for potentially confounding variables such as age, hormonal influence and previous hysterectomy, demonstrates the need for training in proper in smear collection, preparation and examination. Furthermore, this points to the need for constant monitoring, which is critical in providing women with the benefits of cervical cancer screening and in avoiding wasting resources and the adverse effects of false-negative results.

The positivity index, which expresses the frequency of atypical cells in satisfactory exams, was lower (2.2 %) than the 3.0–10.0 % expected by Brazilian Guidelines [19]. Other studies conducted in the country, in Goiás (6.1 %) [17] and in Rio de Janeiro (6.8 %) [12], showed values above 6 %. However, in Maceió only 1.1 % of tests were abnormal [12]. In Spain, the percentage of cytology positivity (any atypia) increased from 3.0 % in 2008 to 3.5 % in 2011 [22]. In countries like England, the United States and Norway, which have well-structured cytological screening programs, the positivity rates were 5.9 % [23], 4.3 % [24] and 5.7 % [25], respectively. Recently, liquid-based cytology specimens from 46,887 women ≥ 21 years of age from 61 centers in 23 states across the United States were evaluated in the ATHENA trial, and the overall cytological abnormal rates ranged from 3.8 to 9.9 % [26].

The HSIL+ percentage of 0.3 % proved consistent with that observed in 2010 in Brazil (0.3 %) [18]. In a previous study, the prevalence of cervical cytopathological lesions was significantly different between Brazilian regions. While no objective information can justify this difference, some authors hypothesize that there may be a difference in the diagnostic performance of the screening, related to the quality of the Pap smear [27]. In England, the United States and Norway, cervical cancer precursor lesions constituted 1.0 % [22], 0.5 % [23] and 0.8 % [25], respectively. It should be noted that in Rio de Janeiro this percentage was 0.9 %, in contrast to Maceio, where the percentage of HSIL+ was 0.1 % [12].

Considering the high incidence of cervical cancer in the state of Maranhão and the low positivity of cervical screening for any atypia and for HSIL+, one can speculate that suspicious abnormalities were not being identified, which may have led to false-negative results. It is therefore important to evaluate and enhance the monitoring of both the pre-analytical phase of smear reading (mainly collection errors) and the analytical phase (scrutiny and interpretation errors).

Pap abnormal rates lower than those described by other studies may have been influenced by events such as inappropriate sample collection or slide preparation, difficulty in the detection of intraepithelial lesions by the professional as a consequence of suboptimal slide preparation, or high workload routine or inadequate training, resulting in the need for continued education of the professionals involved in all stages of screening.

The natural history of cervical cancer was replicated in the results found for the average age of women according to cytological atypia in squamous cell diagnoses, demonstrating its evolutionary character, with an interval between LSIL and HSIL of 12.8 years and between HSIL and SCC of 10.8 years. The classical model of the natural history of cervical carcinoma proposed by the World Health Organization more than 25 years ago estimated that time to progression of grade I and II cervical intraepithelial neoplasia to grade III intraepithelial cervical neoplasia is 3–8 years, while it would take 10–15 years between grade III cervical intraepithelial neoplasia and microinvasive carcinoma [28]. To that end, the analysis of stratification by age group, considering the range of 25–64 years as a reference, identified a statistically significant increase in LSIL detection before 25 years of age (OR = 1.5) and in cervical cancer and precursor lesions in the >64-year old group (OR = 2.8). These results confirm what was already expected, that is, before 25 years of age LSIL prevails, but regress spontaneously in most cases [29, 30]. On the other hand, in women over 64 years, the incidence of cervical cancer and precursor lesions probably reflects the lack of previous cytology and/or poor quality of the previous screening.

In fact, the positive preventive effect was demonstrated clearly in women who had undergone screening at least once among those aged 25–64 years and those aged ≥65 years, who had, respectively, a 10.0 % (OR = 0.9) and a 20.0 % (OR = 0.8) lower chance of having cervical cancer and precursor lesions. This trend continued regardless of age, as the prevalence of cervical cancer and precursor lesions was also significantly lower in women who reported having previous cervical screening. In other words, not having been screened, especially in the >25 age group, significantly increased the chance of having cervical cancer and precursor lesions. Thus, we can hypothesize that deficiencies in the coverage of screening in the state of Maranhão’s population has resulted in high cervical cancer incidence rates.

However, it is noteworthy that prior screening did not increase the chance of detection of LSIL. To better explain this occurrence, the quality of examinations performed, their frequency and the follow-up of women would need to be investigated. It may be inferred in the meantime that this could partly be due to the fact that it is a transient and self-limiting form of colpocytological change, which is expected to exhibit spontaneous regression in most cases [29, 30]. Another possibility might be the limitations of the exam itself, which is affected by the low rate of positivity and lack of representativeness of TZ epithelia found in many cases.

Cervical cancer incidence is inversely associated with HDI, as it is the most common cancer in low-HDI regions and is the cancer with the highest incidence and greatest mortality in women in several countries with medium and low HDI [8]. Similarly, the association between MHDI and the presence of cervical cancer and precursor lesions in Maranhão state was significant. The prevalence of these lesions also demonstrated a tendency to increase as MHDI decreases. Thus, municipalities with medium or low MHDI and with very low MHDI had 40.0 and 260 % more detected lesions, respectively, than municipalities with high MHDI. The state of Maranhão has considerable variation of MHDI among its municipalities, with rates ranging from 0.443 in the municipality of Fernando Falcão (the second-lowest of Brazil) to 0.724 in the capital São Luís [9]. These variations reflect existing differences related to access to health services, education and income distribution, i.e., the quality and living conditions of the population in these geographical areas.

In samples in municipalities with very low MHDI, there was higher TZ representativeness, of the order of 80.0 % as opposed to 59.1 and 42.2 % found in municipalities with medium or low MHDI and in municipalities with high MHDI, respectively. This fact suggests a higher rate of cervical cancer and precursor lesions, which is presumed to stem from better technical preparation of the professionals performing the smear collection, as these are municipalities with fewer health units and lower treatment volumes.

Similar studies of HDI association were not identified, which precluded comparison with the results of this study. However, HDI was associated with cancer prognosis (measured by years of life lost) in a study sponsored by the International Agency for Research on Cancer (IARC) involving 184 countries [31]. An increase of 0.2 in HDI was associated with a 20 % decrease in the risk of developing cervical cancer and a 33 % decrease in the risk of dying from this type of cancer [32]. Another study involving 28 countries of the Pan American Region showed that HDI was inversely associated with the incidence of and mortality from cervical cancer [33]. An ecological study assessed the association of cervical cancer screening rates (Pap smear) with the HDI in the Brazilian state capitals and Federal District. The results pointed towards a positive correlation between HDI and having a Pap smear any time in life or in the previous three years (*r* = 0.66 and 0.71, respectively) [34].

Despite the profile of economic equity that can be intuited between women seen within the public health system in the state of Maranhão, this spatial delimitation by MHDI allowed a more specific demonstration of a strong social inequality regarding the chances of detecting cervical cancer and precursor lesions. Women living in regions with fewer essential quality-of-life conditions, or even devoid of them, may have had greater susceptibility to cervical cancer risk factors. On the other hand, the results also clearly indicate that early cytological diagnosis of atypia depends not only on human development conditions but also on screening quality. This is what was found in municipalities with very low MHDI, which had better screening performance indicators, which can be inferred from the lower percentage of unsatisfactory cytologies and the higher percentage of TZ representativeness in the samples. In that regard, the association between the high quality of population screening programs and the decrease in incidence of and mortality from cervical cancer is supported [35], and this outcome might be projected for the coming decades with improvement in quality life in poor countries [8].

This study has some limitations that must be considered in order to properly interpret its findings. First, a large secondary database was analysed, with more than one million cases, and data were collected over six years from numerous health professionals, in different cities of the state, which may have compromised the quality of information and adherence to the PNCCCU protocols. Important information such as the education level and socioeconomic status of the women were not available or were seldom complete and were excluded from the analysis. In addition, the absence of a unique identifier for the women may have allowed a single woman to be included more than once in the study, preventing exact measurement of the annual prevalence of cervical cancer and precursor lesions in the population. However, the percentage of smears with cervical cancer and precursor lesions may be used as a *proxi* capable of helping in the planning of disease control measures and attracting women who would otherwise not obtain a test. Furthermore, the populational nature of the study, its size and the internal consistency of the observations are strong points to consider.

The findings of this study has as its greatest implication the identification, for the first time in a Brazilian state, the role of MHDI in the pattern of cervical cytological abnormalities (CCAs). When considering the MHDI as a variable, we can even understand specific peculiarities and differences of this association within the same State. Our results certainly will provide subsidies to better qualify the screening program for cervical cancer in that State.

The incorporation of MHDI in this study has identified in its results the existence of inequity in the secondary prevention of cervical cancer even in a poor Brazilian State. Such evidence shows the need to incorporate strategies that stimulate socioeconomic development of the counties in a shared way with the Public Health policies providing an organized and qualified cytological screening.

## Conclusion

This analysis enabled better evaluation of the performance of the National Program for the Control of Cervical Cancer’s actions in the state of Maranhão and can contribute to the recommendation of local actions. The association between MHDI and the occurrence of CCAs and HSIL+ shows that more developed areas with more effective health services have a lower prevalence of these lesions. Failed collections underscore the need for constant and systematic monitoring of actions aimed at achieving high-quality screening, which seems not to be occurring despite an annual average of 227,282 samples collected by the program. To increase the TZ representativeness and positivity index of samples, it is imperative that investments be made in training and skill-building for proper sample collection and analysis. Despite being an opportunistic program, it is essential to promote strategies for managers’, health professionals’ and users’ adherence to the guidelines recommended by the Ministry of Health. Another aspect to be considered is the formulation of policies to reduce social inequality and improve the availability of health services aimed at promoting well-being and social justice, as this would result in the reduction of disparities in regard to access to cervical cancer control strategies.

## Abbreviations

AGC, atypical glandular cells; AIS, adenocarcinoma in situ; ASC, atypical squamous cells; CCAs, cervical cytological abnormalities; CI, confidence interval; HDI, Human Development Index; HPV, human papillomavirus; HSIL, high-grade squamous intraepithelial lesion; HSIL+, high-grade squamous intraepithelial lesions or worse; LSIL, low-grade squamous intraepithelial lesion; MHDI, Municipal Human Development Index; OR, odds ratio; PNCCCU, Programa Nacional de Controle do Câncer de Colo do Útero (Brazilian Cervical Cancer Early Detection Program); SCC, squamous cell carcinoma; SISCOLO, Sistema de Informação do Câncer do Colo do Útero; TZ, transformation zone
